# A battle in the hive against the Queen Bee: reaction of female subordinates’ unconcious mind

**DOI:** 10.3389/fsoc.2025.1554275

**Published:** 2025-07-01

**Authors:** Elif Baykal, Sevil Surucu

**Affiliations:** Istanbul Medipol University, Istanbul, Türkiye

**Keywords:** female leaders, Queen Bee Syndrome, prejudge, unconcious workplace hostility, unconcious thinking theory

## Abstract

When women in leadership roles act antagonistically toward female subordinates, it’s referred to as the Queen Bee Syndrome. Though it often appears as a top-down dynamic, little is known regarding possible subordinate blowback. With the goal to look into the unconscious reactions of female subordinates performing under female leaders in male-dominated workplaces, this exploratory study utilized a qualitative method. Nine female professionals from an array of industries took part in semi-structured interviews, and MAXQDA was employed to assess the data using both inductive as well as deductive content analysis. Preference for male leaders, perceived difficulties with female managers (such as meticulousness and emotional reactivity), divergent views about female leadership, and the effect of social expectations were the primary four themes that emerged. Findings show that subconscious biases against female superiors may be prevalent among female subordinates, that are comparable to the behaviors typically linked to Queen Bee Syndrome. The “Worker Bee Syndrome,” a reversal dynamic in which workers show bias against female leaders, is introduced in the study. The significance of resolving entrenched biases and workplace gender imbalances is made apparent by these bilateral tensions, which raise doubt on assumptions of unidirectional workplace enmity.

## Introduction

The dynamics of business life have begun to change significantly for women, and women have begun to play an active role in business life, progressively gaining the ability to reach positions that they could not previously obtain (Narcikara, 2018). For decades, women professionals have struggled to find a fair place in the workplace (Padavic and Reskin, 2002; Baykal, 2023). In reality, women have always had to labor in more difficult and demanding environments than men. For example, women typically earn less than men in comparable roles and have fewer possibilities to obtain important and managerial positions (Peck, 2021). In addition, they wear them out in several spheres of life as wives, mothers, and workers (Yildirim and Eslen-Ziya, 2021). Despite the fact that the labor force participation rate of women has increased significantly in recent decades (Achhnani and Gupta, 2022), women’s advancement is limited to the lowest levels of the labor market. However, their disadvantageous position remains. Because business life is still a male-dominated jungle, past studies have revealed that women who have held positions of leadership in male-dominated commercial organizations are more likely to embrace gender stereotypes (Cibibin and Leo, 2022). Although it is widely assumed that men in positions of power have prejudices toward women (Garcia-Retamero and López-Zafra, 2006; Begeny et al., 2020; Tabassum and Nayak, 2021), a recent study indicated that women in managerial positions had a higher negative attitude toward female subordinates (Faniko et al., 2016).

On the one hand, past research has found that women in male-dominated workplaces are more inclined to suppress some of their views and behaviors in response to double standards and double binds (Mavin and Yusupova, 2022). Individuals judge the identical actions of men and women differently, according to Foschi (1996) double standards hypothesis; what is considered normal and proper for one may not be equally so for the other. This idea is supported by the fact that, while the number of women in business has increased in recent years, a comparable trend has not been observed in managerial jobs (Cibibin and Leo, 2022). When the existing literature is closely analyzed (Gregory, 2003; Childs, 2012; Bobbitt-Zeher, 2011; Malos, 2015; Kato and Kodama, 2018; Bader et al., 2018; Batool, 2020; Braddy et al., 2020; James et al., 2023), it is clear that gender discrimination by men is one of the most significant causes of women’s underrepresentation in the business environment, particularly in senior positions (Faniko et al., 2016, p. 903). In real terms, women have an advantage in business because they are more adept at creating and maintaining social connections. They are also skilled at controlling and influencing emotions, despite the fact that in workplaces with a predominance of men, this is frequently disregarded.

Women’s difficulty in achieving positions of authority may be significantly explained by the relationship between power and masculinity, which makes it difficult for women to associate their feminine gender with roles that are dominated by men (Cibibin and Leo, 2022). Expectations that female employees will create a solidarity environment and show more supportive attitudes toward one another may be raised due to the negative effects of a predominantly male-dominated work environment on women; however, these expectations may not always be realized in reality. In fact, a lot of female workers would argue that their female supervisors undervalue, ignore, or even hinder them. To put it another way, rather than supporting their fellow female workers, female professionals may take on intrusive behaviors that impede, unnerve, and threaten them—a practice known as mobbing (Narcikara, 2018). Interestingly, previous research has found that female professionals who have achieved certain successes or positions in a male-dominated business environment consciously avoid and distance themselves from their same sex, making life more difficult for them in the workplace (Sheppard and Aquino, 2013; Narcikara, 2018). Several studies conducted in the workplace have previously shown that women are less supportive of other women’s successes than males are (Achhnani and Gupta, 2022).

Previous research has found that women who have held positions of leadership in male-dominated business organizations are more likely to embrace gender stereotypes (Cibibin and Leo, 2022). Because of patriarchal culture, women in high positions in organizations are more critical of their female colleagues, they mostly attribute their professional success to their own merits and prefer to surround themselves with more men than women (Grangeiro et al., 2022). However, according to Maghni and Afilal (2023), women in inferior positions expect their female employees to be more understanding, caring, and responsive to their needs. When this is not the case, they start acting aggressively.

Generally, women are expected to comply with cultural standards by participating in communal and collaborative activities (Kark et al., 2020). Because they hold their managers to higher standards, female employees find it challenging to accept and interact with them as superiors. Male employees do not have the same expectations as female employees, who naturally anticipate their female employers to be more compassionate, understanding, loving, and forgiving (Mavin, 2006, p. 267). In other words, instead of assisting other female employees, female employees may adopt troubling attitudes that obstruct, make them unpleasant, and intimidate them, i.e., they may engage in mobbing. According to Vara-Horna et al. (2022), business involvement in the prevention of gender violence is a more complex process than expected, requiring a reinforced strategy aimed at overcoming managers’ implicit resistance. In this exploratory study, we attempted to demonstrate same-sex psychological violence among women professionals and the potential reverse effect of queen bee phenomena on the part of the subordinates, which is also a form of violence that needs to be challenged.

Vara-Horna et al. (2022) assert that the process of including businesses in the prevention of gender violence is more complicated than one may anticipate and calls for a strengthened approach meant to get beyond managers’ tacit opposition. Despite occasionally being exposed to female leaders, we contend in this paper that when female employees find themselves in male-dominated workplaces, their unconscious minds set off an unconscious thought process that causes them to begin using strategies to defend themselves against the leader and the masculine environment. A useful tool for comprehending the function of executive, intelligent action control in the fluidly automated performance of expertise is unconscious thinking theory (UTT) (Ivy, 2023). In accordance with the “unconscious thinking theory” proposed by Dijksterhuis et al. (2016), we propose that intelligently automated reactions of female subordinates to masculine female leaders originates from unconscious cognitive processing that has coded that ‘masculine’ is the hostile. We tried to demonstrate same-sex psychological violence among female professionals in this exploratory study, taking into account the potential reverse effect of the queen bee phenomenon. We also attempted to demonstrate the reverse effect of Queen Bee Syndrome on the part of the subordinates, which is another form of violence that needs to be challenged.

### Queen Bee Syndrome

The concept of the queen bee, which is the subject of this study, is that female professionals who have achieved certain successes or positions in a male-dominated business environment consciously stay away from their peers and keep a distance from them, making life more difficult for them in the business environment (Narcikara, 2018). Queen Bee Syndrome is a form of perceptual shift in the female mind created by a male-dominated culture. A woman assumes that as long as she represents the dominant gender in society, she will not encounter challenges such as a glass ceiling, will not be taken for granted, will not be regarded insufficient, and will not face snubs for the job she does. However, this is not always enough to achieve the intended outcomes, namely, becoming a person who competes on equal terms with men, is accepted by the dominant male group, and his standing is normalized (Narcikara, 2018).

Powell and Butterfield (2003) claim that women’s desire to exhibit feminine traits conflicts with the demands of their managerial roles, which need a greater degree of masculine traits. The Queen Bee Syndrome gives gender inequality legitimacy and allows it to continue. According to Hussain (2022), females exhibiting traits of a queen bee are more prone to hinder their subordinates’ career advancement and to be resistant to sharing their skills and information. The queen bees are more likely to reject persistent discrimination that upholds the gender hierarchy between men and women, adapt to masculine organizational climates, and legitimize the derogatory and inferior view of women as more emotional and less ambitious employees than men (Derks et al., 2016, p. 463). Similarly, in the Queen Bee Syndrome, while the female manager is struggling with carrying a female social identity in business life (Del Carpio and Guadalupe, 2022) and is concerned about proving herself to the dominant male social group and being accepted by them (Wright, 2016; Bidges et al., 2020), she can keep her fellow female employees away from herself (Gomes Neto et al., 2022). However, this circumstance does not always necessitate the woman boss becoming ashamed of her female identity and abandoning it (Kark et al., 2020). In other words, it might be a circumstance in which she can safeguard her identity as a woman, be proud of it, and again keep herself different from other women (Arvate et al., 2018). According to Baykal et al. (2020), in a workplace where men predominate, the queen bees isolate themselves from the disadvantaged group—women—and, in a sense, take themselves under protection, elevating themselves to the status of unique members of their community.

Derks et al.’s (2016) approach to Queen Bee Syndrome was generally adopted in our study, and according to this approach, queen bees: (1) exhibit masculine rather than feminine traits, (2) physically and psychologically distance themselves from other women, and (3) accept and support the existing gender hierarchy (Narcikara, 2018). According to this theory, certain successful women in business are less inclined to include other women as subordinates in their respective groups, preferring qualified women candidates and women in high-ranking roles (Sheppard and Aquino, 2013). In other words, they do not invest in lower-level employees. Queen Bee Syndrome, according to this opinion, occurs when individuals have a low gender identity with their reference group. In contexts with gender imbalance, this lack of gender identification is obvious (Arvate et al., 2018). The dominance of gender stereotypes in the workplace is a critical element hindering women’s professional growth and forcing top-level female supervisors to be unfriendly to female subordinates (Arvate et al., 2018).

One of the most plausible explanations for Queen Bee Syndrome, according to Klaus (2009), is the scarcity argument, which claims that women have a limited number of executive posts accessible to them and must fight for them (Wuertele, 2017). On the one hand, inadvertent undermining assumption is one of the most common causes of Queen Bee Syndrome. It contends that women leaders may avoid displaying bias toward other women for fear of receiving lower evaluations and even being penalized for fostering diversity (Zhao and Maw-Der-Foo, 2016).

Numerous studies in the literature demonstrate that Queen Bee Syndrome is detrimental to people. Sterk et al. (2018), for example, explored whether Queen Bee Syndrome caused anger, despair, and anxiety, and whether higher levels of Queen Bee Syndrome increased employee intentions to resign. Furthermore, Queen Bee Syndrome can diminish female subordinates of female managers’ level of person-organizational fit in organizations (Baykal et al., 2020). Actually, being in sync with one’s organization is what person-organization fit is all about (Baykal, 2019), and Queen Bee Syndrome may disturb that sync. Individuals who do not receive assistance from their female superiors may risk exclusion and dissatisfaction with their organization.

Actually, the medallion has another feature. Women who worked with a male superior, for example, reported higher levels of well-being (i.e., fewer negative physical symptoms and less distress) than women who worked with a female superior or in gender-mixed superordinate contexts (i.e., with one male and one female superior). Men reported the highest levels of well-being when working in a gender-mixed superordinate team (Schieman and McMullen, 2008). This may offer support to the notion that when women are managed by other women, they perceive their management style as less real and hence more harmful to their well-being.

### Worker Bee Phenomenon

The term “Worker Bee Phenomenon” describes situations in which women at the bottom of the hierarchy reject, alienate, and disparage more senior women in a company (Kark et al., 2020). The existing literature provides empirical evidence that suggests women may detest and negatively assess the interpersonal traits of women who succeed in traditionally “masculine” roles. These studies demonstrate that both genders hold women leaders in lower regard and rate them more negatively on interpersonal measures (Phelan et al., 2008) than they do male leaders (Vial et al., 2016). According to earlier studies, women are more likely than men to disparage senior women because they make junior women feel more threatened as a group and as competitors (Gabriel et al., 2018; Sheppard and Aquino, 2017). Supporting the view regarding existence of worker bee syndrome, Gabriel et al. (2018) empirically revealed that Gabriel et al. (2018) in which women reported experiencing more incivility from female colleagues than from male colleagues which can be explained as a result of women’s limited access to organizational resources which increases competition among women.

As to extant literature, women in male-dominated organizations support the status quo by turning against other women, ignoring derogatory remarks about them, and by being disloyal to them (Nieva and Gutek, 1981). Similar to this, Mizrahi (2003) highlights how a male-dominated workplace devalues women and encourages rival female competitors to disdain one other’s accomplishments, including their own female supervisors. This also affects their preferences about their manager’s gender. For instance; in a study conducted in Turkey, which involved 2,833 participants—1,473 men and 1,360 women—about the preferences of female subordinates regarding the gender of their leaders, the findings showed that 60% of women said they wanted to work with men, 28% said they wanted to work with women, and 12% said they were neutral (Cevher and Öztürk, 2015). Actually, extant literature supports the view that worker bee syndrome can be stemming from same-sex jeaolusy. For instance; in her study, Netchaeva (2014) has shown that envy makes women respond more violently than it does in men, particularly in professional relationships where the two parties are of the same gender rather than the opposite.

On the one hand, when female subordinates are exposed to Queen Bee Syndrome, they want to remove themselves from queen bees (Kremer et al., 2019). Furthermore, they establish a collective and unconscious mechanism to fight queen bees’ unpleasant and unsupportive behavior. Instead of unsupportive queen bees, they portray themselves as supportive leaders of any gender. Furthermore, they are more strongly identified with their own female identities, which compensates for their negative encounters with Queen Bees that exhibit inconsistent femineity (Kremer et al., 2019). The desire to be a good group member may make female subordinates more bonded to one another, leading to a stronger commitment to the feminine role. Feeling psychological ownership for a social group may make female subordinates attach to that social group (Narcikara, 2017) and they tend to distance themselves from the Queen Bee and create problem for the female leader.

The term “Worker Bee Phenomenon” describes situations in which women at the bottom of the hierarchy reject, alienate, and disparage more senior women in a company (Kark et al., 2020). Actually, the term “Tall Poppy” refers to people with remarkable abilities or characteristics in Australia. According to Tall Poppy Syndrome, people associated with tall poppies regularly attack, degrade, or otherwise reduce them to the level of the general public. There is a common belief that anyone who appears to represent success, tremendous talent, or attractive characteristics should be attacked, denigrated, and degraded to mediocrity (Mancl and Penington, 2011). Regarding Worker Bee Syndrome, in workplaces where men predominate, female subordinates may find the hyperbolic trend of the female manager upsetting, and they may perceive her as unsettlingly arrogant and conceited, which contributes to the occurrence of Worker Bee Syndrome.

## Methodology

### Research design and pattern

In this study, a qualitative analytical technique and case study pattern were utilized to examine and comprehend the current status, obstacles, and viewpoints of female subordinates working under masculine female leaders. Qualitative studies are defined by Creswell (2013) as “research in which qualitative data collection methods such as observation, interview, and document analysis are used and a qualitative process is followed to reveal perceptions and events realistically and holistically in their natural environments.” On the one hand, Yin (2009) states that the “case study pattern” should be followed while assessing a problem within the environment or the real-world setting. The pattern of case studies is described as “a qualitative approach in which the researcher collects detailed and in-depth information about real life, a current limited situation, or multiple classified situations within a given period of time, presenting a situation.”

In a qualitative study, a well-defined and well-supported conceptual framework is essential for a research topic to be both clear and focused. Appropriate research methods are chosen to decrease researcher bias and increase trustworthiness, which are characteristics of qualitative approaches. Rigor depends on researcher reflexivity, which is essentially the researcher’s awareness of their own prejudices and justifications for decisions made during the course of the study (Johnson et al., 2020). In this study, we considered Glassick’s Criteria for Assessing the Quality of Research Papers. Hence, first of all, we set a clear purpose for our research. We wanted to understand whether Queen Bee Syndrome is a reality, and in case negative attitudes of women towards women are a two-way situation, we wanted to investigate how and why these attitudes occur. Secondly, we had adequate preparation. We scanned the relevant literature in detail. Thirdly, we used the appropriate research methodology. In this study, we had to make an exploratory study to gain an in-depth understanding of the inherent nature of Queen Bee Syndrome, so a qualitative study was a sound choice (Levitt et al., 2018). Fourthly, we achieved notable outcomes that add to the relevant body of knowledge. In fact, the Queen Bee Syndrome is mostly associated with the emergence of a female leader who isolates herself from other female colleagues in order to achieve important global positions in a male-dominated workplace (Grangeiro et al., 2022; Raja and Riaz, 2022). Our research, however, added to the body of knowledge by demonstrating how followers might have prejudices against their superiors and foster a bad opinion of their female leaders. As a fifth consideration, we presented our findings so that future scholars studying queen bee phenomena might build on them and undertake their own studies. Furthermore, when evaluating the data and presenting the findings, we made an effort to be as impartial and objective as possible. Robust descriptive language combined with thick and detailed descriptions to give enough context allows the reader to assess the descriptions’ reliability, believability, transferability, and confirmability. In qualitative research, rigor is defined as making sure that the methodology, findings, and research design are clear, accessible, repeatable, and devoid of bias (Leung, 2015). In this study, when designing our research we scanned the related literature in detail to make sure that we have profound knowledge about the related field. Furthermore, when designing the research and when creating research questions, we get consultancy from academicians having interest in the research field and female managers having considerable professional experience to ensure rigour of the study. Moreover, during the interviews we used two people from our research team to make sure that the interviews are rigorous and free of bias. Furthermore, when analyzing and reporting the data we made our codes and analyzes checked by senior researchers who has profound experience in qualitative studies. Besides that two pilot interviews were conducted to test the comprehensibility of the interview questions and the validity of the questions were confirmed by sharing the results of these interviews with experienced researchers and experts from professional business life.

### Important terms

Queen Bee Syndrome: Queen Bee Syndrome refers to the tendency of some women in higher positions, especially in male-dominated jobs, to distance themselves from their fellow women and not support them.

Worker Bee Syndrome: Worker Bee Syndrome refers to the tendency of female staff working as subordinates to distance themselves and act negatively from female managers at higher levels, especially in male-dominated jobs.

### Sampling for interview participants

In this study, the sample was created by criterion sampling, which is one of the purposeful sampling methods. Purposeful sampling envisages selecting situations that are rich in information about the subject of the research in order to conduct deep and detailed research on the subject. In criterion sampling, which is one of the purposeful sampling methods, the sample is composed of people, events, and situations that have characteristics related to the research subject (Büyüköztürk et al., 2012). In the study, white-collar female professionals working in the service industry and working under female leaders were selected as a sample. Within the scope of the research, Professional women from nine different companies were interviewed between May 12, 2023, and August 19, 2023. Three of these interviews were conducted online. The reason for conducting these interviews online was because the participants indicated that they would feel more at ease in an online setting. Semi-structured guide questions were used during the interview. The structured part of the questions composed of 11 preplanned questions investigating possibility of Queen Bee Syndrome and Worker Bee Syndrome. We asked questions as “*Do you think there are significant differences between female managers and male managers?. If you had a choice, would you like to work with a male or female manager? Why?. Do you think female managers differ from male managers in terms of success in managerial positions? In your opinion, what are the characteristics of successful female managers?”*

When the structured questions do not provide enough context to foster a deeper understanding of the structured questions, the unstructured questions are intended to be utilized as follow-up inquiries in accordance with each interview’s flow. The interviewer gave an explanation of the interview’s goal before it started. Participants were told about anonymity, the fact that their participation was entirely voluntary, and their freedom to withdraw from the study at any moment during the focus group. Before each participant’s verbal assent, the nature and goal of the study were described to them. Prior to the interviews, permission to audiotape the session was obtained orally. The interviews took place in a peaceful, comfortable location.

To maintain confidentiality, participants are assigned letters such as Y, B, G, H, F, J, M, C, and T. In online interviews, the participants answered 11 semi-structured questions, which were videotaped with their permission. The interviews lasted between 25 and 40 min on average. First, the written interviews were reviewed for content, and themes and codes were developed. The study used “content analysis,” which is described as “a family of research techniques for making systematic, credible, or valid and replicable inferences from texts and other forms of communication” by Drisko and Maschi (2017). First and foremost, research data were generated in this setting by transcribing audio recordings and using the notes made. The primary goal of the study and the content analysis were then taken into consideration when classifying the data. Afterwards, the data were examined and categorized using MAXQDA 20. Maxqda is a well-known software application for evaluating qualitative and mixed-method data. Maxqda can evaluate text, photos, videos, tweets, and focus group discussions, among other data types. The codes in the themes were decoded, and samples from the interview materials were used to support the statements.

### Charachteristics of participants

Before developing interview questions, a thorough assessment of the literature was conducted. Two preliminary interviews were conducted to test comprehension of the interview questions. The findings of the pilot interviews were shared with an academic specialist in the field, who confirmed that the questions were correct. When we first started preparing our interview questions, we wanted to ensure that they were sound and helpful in understanding the subject matter of our study. To that end, we wanted to have management and an academician oversee the questions. The coding audit mechanism that assures internal consistency requires at least 80% agreement among coders (Patton, 2002). The coders reached an agreement of 87.50 percent.

As previously stated, we interviewed 9 people. As mentioned before the participants are female professionals from service industry that are working as subordinates of female leaders. In Table 1 you can find the charachteristics of participant below.

**Table 1:** Participants charachteristics.

Participants	Age	Sector	Title
M.	35	Health	Customer Relations Manager
F.	44	Communication	Technician
H.	29	Law	Lawyer
Y.	25	Education	Secretary
G.	34	Education	Laboratory Worker / Biologist
T.	39	Education	Secretary
B.	52	Finance	Manager
J.	45	Bank	Team Leader
C.	40	Education	Assistant Director

In an exploratory study, a comprehensive depiction of every facet of the phenomena under investigation is not our goal. When a study presents fresh perspectives that significantly advance or refute existing knowledge, we are typically satisfied. In qualitative research, sample adequacy, data quality, and variability of relevant events are often more important than the number of participants (Malterud et al., 2016). The sample size is determined by taking into account the diversity of the research data and the sources used to understand the event, subject or phenomenon (Creswell, 2017, p. 109). Creswell reported that the number of participants between three and 10 is sufficient for studies with a phenomenological qualitative design (Creswell, 2013, p. 189). This situation, where various data cannot be obtained from the participants in the research and the data becomes repetitive, is called the “saturation point” (Creswell, 2017, p. 111). Actually, the reason we stopped at nine interviews was that during the final three, we began to receive identical responses to the same topics. When the interviewees’ responses become repetitive and saturating, it is advised to halt the interview process in qualitative research (Hennink et al., 2017). These participants were professional female workers who worked for female leaders. As a result, our sample was appropriate for determining whether they were subjected to Queen Bee Syndrome or experienced Worker Queen Bee Syndrome.

### Findings of qualitative study

Data obtained during the field study from the interviews were analyzed using the deductive and inductive content analysis method (Elo and Kyngs, 2008). The transcripts were read to derive “meaning units” (words, phrases, or paragraphs) based on interview question topics, and they were modified as new information emerged from the narratives. Then, all data were reviewed for content to generate themes, new codes, or modify the induction codes. To enhance credibility, we selected participants from different ethnic organizations, departments, and age groups. To assess dependability, peer checking by another team member to re-analyze some of the data was performed. The interviews were transcribed and read several times to gain a thorough understanding of the entire conversation. For transferability, a detailed analytical description of the context, methodology, and limitations, as well as maximum variation sampling, were provided. When the interview findings were coded in the maxqda algorithm, we discovered 14 distinct codes, which are detailed in Table 2. According to the table, the most commonly stated topics are “it is easy to work with a man,” “female managers are meticulous,” “female managers are tough,” and “women act with their emotions, cannot be objective.”

**Table 2:** Code list.

**Themes**	**Codes**	**A**	**B**	**C**	**D**	**E**	**F**	**G**	**H**	**I**	**J**	**K**	**L**	**M**	**N**	**Total**
Theme 1	It’s easy to work with man (A)	0	3	4	2	3	2	1	4	1	1	2	3	3	2	31
Theme 2	Female manager is meticulous (B)	3	0	3	2	2	2	1	3	0	1	2	3	3	1	25
Theme 2	Jealousy (C)	4	3	0	1	3	1	2	1	1	1	2	2	1	1	23
Theme 4	Preferring women in social relations (D)	2	2	1	0	1	1	1	2	0	1	3	2	3	1	20
Theme 2	Women are inconsistent (E)	3	2	3	1	0	2	1	1	1	1	1	1	0	1	18
Theme 1	Preferring a masculine female manager (F)	2	2	2	1	2	0	1	0	0	0	0	1	0	1	12
Theme 1	Male manager is respectful (G)	1	1	1	1	1	1	0	0	0	0	0	0	0	1	7
Theme 2	Female managers are tough (H)	4	3	2	2	1	0	0	0	1	2	4	3	5	1	28
Theme 3	Appreciating the effort (I)	1	0	1	0	1	0	0	1	0	1	1	0	0	0	6
Theme 3	Feminine female manager (J) preference	1	1	1	1	1	0	0	2	1	0	3	0	2	0	13
Theme 3	Women can be successful managers (K)	2	2	1	3	1	0	0	4	1	3	0	2	4	0	23
Theme 3	Female managers are caring and motherly (L)	3	3	2	2	1	1	0	3	0	0	2	0	3	0	20
Theme 2	Women act with their emotions, cannot be objective (M)	3	3	1	3	0	0	0	5	0	2	4	3	0	1	25
Theme 4	Social pressure on women (N)	2	1	1	1	1	1	1	1	0	0	0	0	1	0	10
	**Total**															

When the code list occurred, we noticed that some important themes occurred in the study. First of all, the codes explaining that women subordinates feel more comfortable with male leaders form the first theme (Theme 1), including the codes: it is easy to work with men (20), male managers are respectful (2), and preferring a masculine female manager (2). The total number of coded parts in this theme is 24. The second theme explains the difficulties of working under a female manager (Theme 2), including the codes: female managers are meticulous (13), female managers are jealous (6), women are inconsistent (5), female managers are tough (12), and women act with their emotions and cannot be objective (10). The total number of coded expressions in this theme is 46. As a third theme, we have collected expressions preferring a female manager (Theme 3), including the codes: female managers appreciating the effort (2), feminine female manager preference (4), women can be successful managers (8), and female managers are caring and motherly (9). The total number of coded parts in this theme is 23. As a last theme (Theme 4), women’s solidarity in social life attracts attention. Some of our participants think women are better friends in social life than business life, which is coded as preferring women in social relations (5), and the codes indicating that there is social pressure on women in general (5). The total number of coded phrases in this theme is 10. As we can see from this code distribution, the codes related to the 1st and 2nd themes stand out more in terms of frequency, and they support each other in terms of meaning. In other words, the number of codes claiming that it is easier to work with men and the number of codes claiming that it is difficult to work with female managers are significantly higher.

## Discussion

Being a woman in many societies poses a number of challenges. Women face unique problems as a result of their gender, in addition to the challenges of daily life. Women face unique challenges in business. In a competitive workplace, women often exhibit aggressive behavior towards other females, leading to Queen Bee Syndrome (Narcikara, 2018). They tend to be masculine in order to be successful in management roles, and they must negotiate masculine standards that would normally exclude them from managerial positions; as a result, they feel obligated to conceal their femininity, and they begin to lose themselves (Battistelli and Mariani, 2011), leading to Queen Bee Syndrome (Narcikara, 2018). This study, like many others (Derks et al., 2016; Wuertele, 2017; Cibibin and Leo, 2022), sheds light on the existence of workplace spirituality. Moreover, this study is among the scarce number of studies conducted in Turkey revealing this syndrome among female preofessionals (Akman and Akman, 2016; Baykal et al., 2020; Mert, 2022) (see Table 3).

**Table 3:** Theme.

**Themes**	**Codes**	Y	B	H	F	G	J	M	C	T	
Theme 1	It’s easy to work with man	3	4	2	3	0	0	0	2	6	20
Theme 1	Preferring a masculine female manager	1	0	0	0	0	0	0	0	1	2
Theme 1	Male manager is respectful	0	0	0	0	0	0	0	0	2	2
Theme 2	Female manager is meticulous	3	1	0	0	3	0	1	0	5	13
Theme 2	Jealousy	1	1	0	1	0	0	0	0	3	6
Theme 2	Women are inconsistent	3	0	0	1	0	0	0	0	1	5
Theme 2	Female managers are tough	0	2	1	3	1	0	1	4	0	12
Theme 2	Women act with their emotions, cannot be objective	0	2	3	0	1	1	1	2	0	10
Theme 3	Appreciating the effort	0	0	0	2	0	0	0	0	0	2
Theme 3	Feminine female manager preference	0	0	0	1	1	2	0	0	0	4
Theme 3	Women can be successful managers	0	0	1	1	3	1	2	0	0	8
Theme 3	Female managers are caring and motherly	2	4	2	0	0	0	1	0	0	9
Theme 4	Preferring women in social relations	0	0	1	0	0	1	2	0	1	5
Theme 4	Social pressure on women	0	0	0	0	0	0	0	3	2	5
	**Total**										**103**

According to Webber and Giuffre (2019), patriarchal organizational cultures stereotype women negatively, resulting in behaviors such as a lack of solidarity among women and leading to queen bee mentality. The rejection of actual gender discrimination by women provides compelling justification for the status quo (Mufti et al., 2021). Long-term exposure to a male-dominated workplace that fosters a masculine culture makes employees gender blind. They learn that in order to succeed in the workplace, they must shed their feminine identity and adopt a dominant set of masculine traits that set the stage for success in environments where men predominate (Narcikara, 2018). For instance; Born et al. (2022)‘s study, which investigated the relationship between leadership and team composition and discovered that even women working female-dominated organizations prefer male leaders whose reasults are parallel to our study revealing that many of the participants in the study admitted that they prefer male leaders rather than female leaders. The Queen Bee phenomenon is the result of cultural conditions that jeopardize women’s social identities in professional business life, specifically the conflict between women’s personal goals and gender stereotypes passed down through generations (Cibibin and Leo, 2022). While women view each other as competitors in non-business domains, they compete more fiercely in the workplace (Benenson and Markovits, 2023), causing female followers to be more hostile to other females, even if she is their leader. While researching how queen bee syndrome affects employees at work, Harvey (2018) discovered that female managers may exhibit aggressive behavior in order to be taken seriously and respected by their subordinates. In our study, which supports Harvey (2018), we discovered that many female subordinates believe their female managers are tough and manipulative, making it difficult to work with them. Moreover, the extremely masculine and unfavorable attitudes of leaders toward female subordinates may also cause female subordinates to form negative opinions about their managers, resulting in the phenomenon known as worker bee syndrome. Actually, worker bee syndrome is caused by the same factors as Queen Bee Syndrome (Baykal, 2023).

### Theme: 1: It is easy to work with men

**Figure 1:**
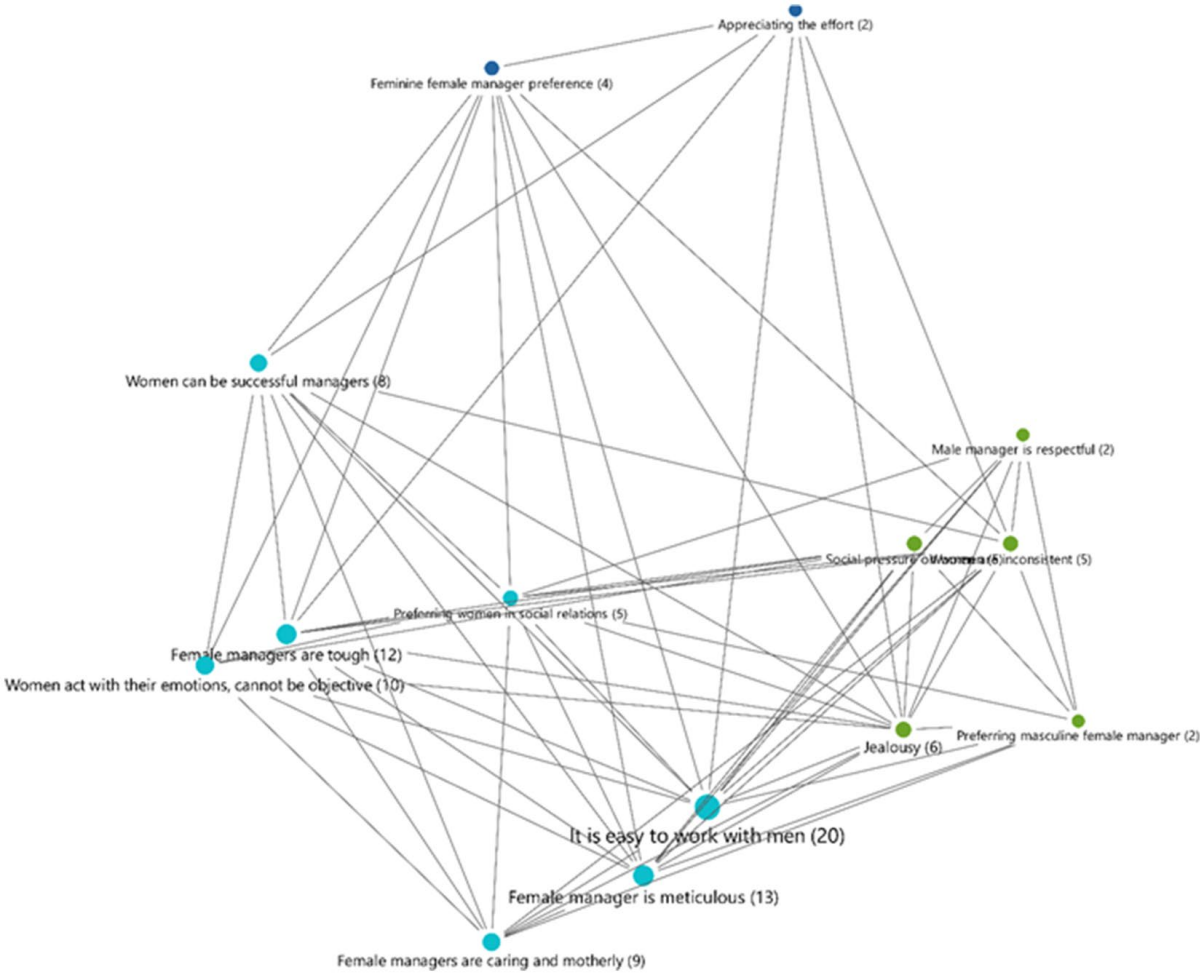
Code coexistence graphic.

According to the facts described in findings part and according to code frequencies described in Figure 1, we can figure out that female subordinates prefer to work with male bosses (Theme 1); this was stated 20 times (by six distinct participants) throughout the interviews. The code “It is easy to work with men” says that female employees often feel more at ease while working with male supervisors since male managers are less detail-oriented and more outcome-oriented. Women feel more comfortable working under male managers. They think that men are more clear and understandable, which makes life easier for them. For instance, Participant F commented about the code, *“He expressed very clearly what he wanted.” “He was telling me exactly what I needed to do, and I accomplished it without any difficulty.”* (F) From the high frequency of this code, we can infer that female subordinates like certainty and clarity in the work environment, which is more common when working under male managers. Moreover, advantages such as a male manager allowing his employees to make their own decisions and interacting directly were also discussed. For example, consider Participant T’s statement: *“I can get along better with a male manager than a female manager. I can be more interactive in my speech. Sometimes I do not comprehend women’s intentions.”* (T) Hence, many participants believe that male managers give them some kind of freedom of speech, and they have higher tolerance. Such perceptions contribute to the general negative perception of female leaders and cause them to lack self-confidence while seeking executive roles. This could be because they perceive female supervisors to be antagonistic (expressed six times), inconsistent (expressed five times), meticilous (stated 13 times by six different persons), and tough (expressed 12 times by six different people). Furthermore, the fact that the majority of participants believe male managers to be courteous may be reinforcing their conviction that working with male managers is easier than working with female managers. The code “Male manager is respectful” includes claims that male supervisors are more distant and cautious with employees of the opposite gender and do not intervene. They treat this difference with respect when they do not fully grasp it. Participant T, for example, stated, *“But because the male manager does not know much about the emotional world of women, he is cautious if he is unsure how to act.”* (T) *“They avoid making comments or acting respectfully on a subject that they do not have control over and do not know.”* (T) On the one hand, many of our participants thought that male bosses were more appreciative. The “Appreciating the effort” code includes words about valuing the employee’s efforts and not dismissing their contributions. At this point, participant F says, *“It was very meaningful that our male manager thanked us in front of everyone, mentioning our names one by one, and said things like he contributed like this, she did this.” (F) “We thanked our male manager for providing us with so much value.” He said, “You do not have to thank me for that.” You’re doing an excellent job, and I appreciate it. But our prior female manager had never done anything like this.” (F)* Furthermore, some utterances are related to the manager’s dismissal of the subordinate’s performance and the assertion that they do not deserve their pay. The following is Participant F’s statement regarding this point of view: *“Our manager was saying things like, Do you truly think you deserve this money? Even spending 8 hours at that desk made me feel like I deserved that money. (F)* These illustrations suggest that a sizable portion of participants think male leaders are more understanding and appreciative.

### Theme: 2: Female manager is meticulous

In relation to the theme 2, the code “female manager is meticulous” actually indicates that female managers are more precise and think in detail. Female managers, according to participants, are more controlling than men, creating an extra burden for female subordinates. For instance, Participant B’s statement about this code is as follows: *“Women need more time to trust. In other words, she feels compelled to exert constant control over the personnel while delegating responsibilities.”* (B) The expressions of participants T and G should also be taken as examples for his code: “*the pressure they apply, the mobbing, and so on. It’s all about the effort they put into performing good work, in my opinion.”* (T) *“When a case is to be decided upon, the woman thinks too much in detail.”* (G) From these statements, we can infer that female subordinates feel disturbed by too much control exerted on them by their female leaders. Furthermore, specifically, the code “Female managers are tough” contains phrases indicating that female supervisors are harsh on female employees and attempt to exert power over them. In relation to this code, some of the participants provided the following explanations: *“she wanted to have power over us, even scare us. It was really hard for us…”* (F) *“…she was like I’ve been through this situation too; you have to work. She said, do not make excuses. It turned into cruelty after a while…”* (G) Female managers, are said to be harsh to their colleagues during the interviews because they feel obligated to show themselves in the professional sphere. In response to this criticism, participant B stated, *“I believe female managers are tougher and more normative.” “They symbolize her tenacity on her team because she had to prove herself a little more.”* (B) On the one hand, some participants believe that the female boss uses severe behavior to establish power over individuals she perceives to be her rivals. The following is participant M’s statement regarding this code: *“women can be the enemy of women. I believe it is due to the competition. She may push the lady she considers a rival…”* Actually these are significant codes supporting the existence of Queen Bee Syndrome. According to these statements we can infer that female leaders are considering their female subordinates as rivals and they are behaving hostile and tough towards them. The code “Women act with their emotions; they cannot be objective” is made up of assertions regarding female managers acting with their emotions, not objectively but impulsively. This code supports the possibility that female subordinates also have negative attitudes towards their female leaders. Hence, Queen Bee Syndrome is a two-way syndrome. Some of the participants stated that *“female managers in general are very reactive. For example, if she becomes enraged over something, she does not think at all*. *Should I act like this right now? Should I not? No, she just reacts right away.”* (H) In a similar way, *“male managers are a little more objective; female managers are a little more emotional.” (M). “Women express their emotions more openly at work. “Women show their emotions more in the work environment. They can get emotional.”* (G) *“If there is a problem among the staff, they can solve the problem among themselves and move forward. But the female manager does not allow this and always intervenes.”* (H) This could be because they believe women bosses are untrustworthy since they view women managers as inconsistent, again supporting the existence of *reverse queen bee syndrome,* which can be explained as the prejudices of female subordinates towards their female leaders. The code “Women are inconsistent” contains comments like inconsistency and unpredictability in female bosses’ behavior and, as a result, faltering or injuring female employees. The following are the statements of participants T and Y. *“Female managers can change the way they work on the road, or sometimes their mood can change depending on their emotional intensity.”* (T) *“They are unable to maintain a balance in terms of behavior and attitude. “As a result, when their behavior* var*ies, it might be detrimental to the employees surrounding them.”* (Y) These remarks can also be seen as evidence of the unfavorable attitudes and presumptions held by female subordinates toward their female bosses, which suggests that the Queen Bee Syndrome is a two-way street.

### Theme: 3: Female managers appreciating the effort

Interestingly, in relation to theme 3, although female subordinates mentioned that women managers are caring (mentioned nine times by four different participants), only three of the participants mentioned that women can also be successful managers. For example, M said, *“I cannot understand why female professionals cannot be in leadership positions; we are as competent as men.”* (M) On the other hand, there are a few phrases that describe female subordinates’ preferences for female managers. For example, the code “feminine female manager preference” includes phrases indicating a desire to work with women who are more feminine as managers. Participants J and F made the following statements: *“I prefer a more feminine, delicate, gentle style.” (F) “I always prefer a female manager who has not moved away from her own identity, who has not become masculine, and whose concern is to produce together.”* (J) The role congruity hypothesis could be said to be at the root of this view. According to Castaño et al. (2019), women are perceived as less competitive when they adopt feminine attitudes and as chilly when they adopt more masculine behaviors. This conclusion also lends credence to the idea of a two-way Queen Bee Syndrome, in which followers detest aggressive, competitive female leaders and have stereotypes about those with more masculine traits. Similarly, individuals who support female managers stated that women make better leaders since they are more humane, caring, and motherly. The code “Female managers are caring and motherly” includes words such as female managers are more caring and helpful to their employees. From this vantage point, women may be better suited for the servant leader role since they share power. This is confirmed by the statements of Participants H and B: *“She is more possessive and concerned with the problems of her employees.” It strengthens the relationship.* (H) *“I believe women are more compassionate than men. Men are more straightforward.* (B) Likewise, *“the male managers like, we can let this person go, it’s fine! We’ll find another. They believe that there are always other potential employees*. *The female manager is extremely protective of her good employee. Because women are possessive, I believe they are more comfortable speaking with upper management to defend their personnel. They are far more determined than the male manager when it comes to defending someone.”(H)* Furthermore, it was stated that women can empathize better than men. As an example, participant Y stated, *“Women’s sense of empathy is much stronger.” “Women are simply stronger. Men are currently weaker.”(Y).*

On the one hand, some participants believe that female leaders can achieve greater success. The code “Women can be successful managers” is made up of sentences that express the notion that women can be better managers than men. Some participants, for example, stated that *“when given the opportunity, female managers achieve much greater success than male managers.”* (J) *“If we are talking about the team’s commitment to the manager, if we are talking about co-managing, I see that the female managers do a better job in my current experience.”* (M) *“I believe that if a woman wants to, she can be a very successful manager. “They have a lot more leeway.”* (F) But these codes are scarce in frequency. This made us feel that these ideas are not shared by most of the female participants.

### Theme: 4: Women’s solidarity

*Theme* 4 is also not very illuminating since there are only 2 codes with weak frequencies explaining women’s awareness about social pressure on them and the need for social support among them. For instance, for the code of social pressure on women, C said, *“We are all aware of the glass ceiling. I believe that rather than losing time, we should help each other.*”(C) Interestingly, we discovered in our studies that, while women favor other females in social contacts, their perspectives shift in business. Actually, the code “Preferring women in social relations” in our study identifies comments in which female employees prefer female managers to male managers in social relations. In this context, some participants’ statements are as follows: *“I prefer women when it comes to expressing my thoughts more easily; the connection between women is different after all.”* (M) *“I get along better with women in social relationships”* (T) *“I prefer to work with a female manager because it is easier for me to communicate with a female manager.”* (H) As mentioned before, the number of participants mentioning this preference is quite limited. However, the code “Preferring a masculine female manager” is more common, and it contains statements indicating that female employees prefer their female managers to be more masculine in their behavior and thinking. The following are the statements of Participants T and Y about this code. *“I prefer a masculine female manager.” (T) “I think women who can think more masculinely are better to work with” (Y)* Moreover, many participants considered that female managers are too emotional to be leaders in professional interactions.

Actually, there are also some self-evaluations regarding women’s position in social life among the explanations of our participants. For instance, “social pressure on women” consists of statements about the various roles that women take in both their private and business lives and the tension or negative behaviors that these bring on them. The statements of Participant T and Participant F are as follows: *“For example, even in a job interview, if you are single, do you have a fiancée?” (T) “Are you thinking of getting married? When hiring, they think that if you are newly married, she will have a child soon and leave. The women are already in a disadvantaged position and may feel heavy. Because, as women, we have a social burden on us.”* (C) As seen in the above examples, in the statements related to theme 4, we can find expressions giving hints about women’s learned helplessness in a male-dominated business world that creates the necessary ground for Queen Bee Syndrome.

As shown in Figure 1, in the code coexistence graphic, it is found that some code coexistences are really significant in revealing the negative attitudes of male subordinates regarding female managers. For instance, there is a strong correlation between the code that indicates “jealousy” and the code that indicates “easy to work with men,” suggesting that professionals who find it easier to collaborate with men also perceive women to be jealous of males. The code coexistence between “easy to work with men” and “female managers are tough” also provide indications of Worker Bee Syndrome. Moreover, the code “female managers are tough” and “Women act with ther emotions, cannot be objective” coexist together which is also a hint for us for the prejudges of female subordinates towars their female leaders.

Additionally, the five times that the codes “female managers are tough” and “women can be successful managers” coexist are interesting because, while some think that women can be successful managers, they also think that this is possible when they are tough, which is another way of saying that they are rather masculine. This also suggests that there is a Queen Bee Syndrome at play, which supports the idea that female professionals in environments that are traditionally masculine think that in order to succeed, they must also exhibit masculine characteristics (Narcikara, 2018). The most common coexistences in our sample group suggest that female professionals prefer working with male managers over female ones and that success in male-dominated work environments is contingent upon masculinity. They find it more convenient and enjoyable to work under male managers.

In line with many other studies (Baykal, 2023; Kark et al., 2023; Holmes et al., 2017), Our findings showed that, in addition to the risk of queen bee disease, there is a high possibility of experiencing Worker Bee Syndrome in male-dominated organizations. Actually, because of the pressure on women to succeed and increase their power, as previously stated, they sometimes prefer to engage in undesirable activities to improve their positions that cause negative reactions on the part of their subordinates (Baykal, 2023). Martin and Phillips (2017) suggest that a potential remedy for Worker Bee Syndrome is for women to become “blind” to gender inequalities. This will boost the confidence of female managers, particularly those who work in industries where men predominate.

## Conclusion

This study illustrates a comprehensive understanding of the relationship between Queen Bee Syndrome and Worker Bee Syndrome. This study confirms the existence queen bee syndrome in the Turkish business environment, where women unconsciously avoid other women and do not support them in male-dominated environments. As to Queen Bee Syndrome, Women feel helpless and oppressed in social societies where men wield cultural dominance, and those who aspire to prominence want to disassociate themselves from their undervalued in-group, often at the expense of abusing and hurting female members of their circle (Ellemers et al., 2004). While designing the study, we predicted that the situation mentioned above could occur in the Turkish business society, which is still quite patriarchal. Many of our participants, during the interviews’ initial stages, either denied or tried to conceal that they are prejudged against their female managers and in later parts of the interviews they admitted that somehow may be unconsciously they fear or dislike working with female managers. Actually, these findings is also parallel with assumptions of unconscious thought theory. According to this theory, when conscious attention is focused elsewhere, object-relevant or task-relevant cognitive or affective thought processes take place (Dijksterhuis and Nordgren, 2006). We refer to this as unconscious thought. Our conclusions indicate that in patriarchal, male-dominated workplaces and societies, women are aware that men wield the majority of power. For this reason, some members of their ingroup, such as their female bosses, make a concerted effort to look like men in order to succeed. Unconsciously, they feel nervous, even if they understand why these queen bees are acting in this way. They find queen bees unpleasant because, unconsciously, they pair these women with the town’s luckier and snobbish qroup. In other words, their too masculine style disturbs them and reminds them of their devalued position in male dominated work atmosphere. The fact that one of their sisters acts like one of the male foes oppressing them disturbs them instinctively. Our research has shown that Worker Bee Syndrome is a prejudice that is at least as significant as queen bee syndrome. As to this syndrome, when exposed to or suspicious of queen bee syndrome, female subordinates get repulsed and begin to overreact to any treatment from their female superiors. On occasion, they overstate their issues with them and even turn antagonistic without a good reason (Ely, 1995). It explains why women in lower positions tend to detest and harbor prejudices against female managers, and why they frequently feel more at ease working with male managers. In essence, both the queen bee and Worker Bee Syndrome can be considered in the category of self-fulfilling prophecy. That is to say, male dominated work environment creates queen bee syndrome and the same environment give way to prejudges against women leaders. This bilateral relationship worsens the ingroup problems among women professionals making things more difficult for both female leaders and subordinates. The majority of participants in our study expressed a preference for working with female managers over male managers, and they also mentioned having unfavorable opinions about female bosses. This led us to conclude that there are prejudices against female subordinates as well. As a result, we came to the conclusion that worker-bee syndrome and queen bee syndrome coexist. Stated differently, Queen Bee Syndrome does not take precedence over the occurrence of Worker Bee Syndrome. In settings where men predominate, this becomes a two-way street where neither party can be certain of what sets off the other.

### Managerial implications

Women’s hierarchical position in official or informal power connections remains problematic in patriarchal cultures like Turkey, despite organizations’ efforts to modernize and embrace a more democratic approach. For this reason, like in the example of queen bee syndrome, they occasionally turn to novel means of securing administrative positions. Many female subordinates become agitated and more hostile toward these so-called Queen Bees who mimic male counterparts after noticing that some female leaders are overly trying to look like powerful men. This leads to a two-way syndrome where relationships between female leaders and members are distorted. This study sheds light on the reciprocal nature of Queen Bee Syndrome, highlighting the fact that mobbing directed against female subordinates can also be redirected from subordinates to leaders. Interventions aimed only at improving perceptions of women’s legitimacy as equal candidates for superior positions will be ineffective. This creates the impression that women are to blame for their negative perceptions and unequal treatment in the workplace. Female professionals who work in firms where the culture considers them less capable of achieving managerial positions than men see this as a threat to their social identity. For those competing for managerial positions, the best thing they can do is act in ways that improve their overall gender status through “collective mobility” by supporting one another rather than viewing one another as potential rivals and foes. This will also be a cure for both Queen Bee Syndrome and Worker Bee Syndrome, both of which promote negativity in the workplace by creating prejudgments against the other. More opportunities for female subordinates to advance can help reduce the issues between female leaders and female members. This will help break the male-dominated environment that is the root cause of both Queen Bee Syndrome and Worker Bee Syndrome. Additionally, it is possible to organize unique events and occasions that can serve as icebreakers by allowing female leaders and subordinates to spend more time together. It is possible to establish mentoring programs that foster enduring, mutually trusting relationships between female managers and female employees. Furthermore, it is possible to develop performance management and punishment systems that prevent mobbing.

### Limitations

This study is a novel exploratory study and tried to explain the coexistence of queen bee syndrome and Worker Bee Syndrome and why they occur. Our study is illuminating in empirically showing the bilateral relationship between these two syndromes. It is the first empirical study, both in Turkish and international literature, emphasizing the two-way nature of Queen Bee Syndrome. Moreover, it is one of the rare studies in the literature mentioning Worker Bee syndrome and empirically revealing existence of it. The fact that the study was conducted only with participants working in the service sector and the limited number of participants creates a limitation in generalizing the explanations resulting from the research. In addition, the fact that only female professionals in subordinate positions were interviewed in the study is another source of limitation.

### Recommendation for future studies

In future studies, a wider range of participants can be reached, and the representativeness of the sample can be increased. The validity of the research can be tested in different sectors. Additionally, Worker Bee Syndrome can be examined in more detail by adding leading female professionals to the study. Comparisons can be made between different cultures.

## Data Availability

The raw data supporting the conclusions of this article will be made available by the authors, without undue reservation.

## References

[ref1] Achhnani B. Gupta B. (2022). Queen bee: the culprit or the victim of sexism in the organisation? Manager Br. J. Admin. Manag. 58, 63–70.

[ref3] Akman G. İ. Akman Y. (2016). Kraliçe arı sendromu bağlamında kadın öğretmenlerin kadın yöneticilere ilişkin görüşleri. Bartın Univ. J. Fac. Educ. 5, 748–763. doi: 10.14686/buefad.v5i3.5000195251

[ref5] Arvate P. R. Galilea G. W. Todescat I. (2018). The queen bee: a myth? The effect of top-level female leadership on subordinate females. Leadersh. Q. 29, 533–548. doi: 10.1016/j.leaqua.2018.03.002

[ref6] Bader B. Stoermer S. Bader A. K. Schuster T. (2018). Institutional discrimination of women and workplace harassment of female expatriates: evidence from 25 host countries. J. Glob. Mobil. 6, 40–58. doi: 10.1108/JGM-06-2017-0022

[ref7] Batool F. (2020). Gender discrimination at workplace and mental health of women: a systematic literature review. PalArchs J. Archaeol. Egyptol. 17, 622–633. doi: 10.1016/j.stae.2023.100057

[ref8] Battistelli A. Mariani M. (2011). Perceived organizational support: validation of the Italian version of the survey of perceived organizational support (with eight items). G. Ital. Psicol. 38, 189–214. doi: 10.1421/34845

[ref9] Baykal E. (2019). Person organization fit: spiritual way to boost performance. Asya Stud. 3, 31–43. doi: 10.31455/asya.597151

[ref10] Baykal E. (2023). Same-sex workplace incivility: women against women. Person. Focus 15, 24–34.

[ref11] Baykal E. Soyalp E. Yeşil R. (2020). “Queen bee syndrome: A modern dilemma of working women and its effects on turnover intentions” in Strategic outlook for innovative work behaviors: Interdisciplinary and multidimensional perspectives, 165–178.

[ref12] Begeny C. T. Ryan M. K. Moss-Racusin C. A. Ravetz G. (2020). In some professions, women have become well represented, yet gender bias persists—perpetuated by those who think it is not happening. Sci. Adv. 6:eaba7814. doi: 10.1126/sciadv.aba7814, PMID: 32637616 PMC7319752

[ref13] Benenson J. F. Markovits H. (2023). Married women with children experience greater intrasexual competition than their male counterparts. Sci. Rep. 13:4498. doi: 10.1038/s41598-023-31816-0 36934175 PMC10024730

[ref14] Bidges D. Wulff E. Bamberry L. Krivokapic-Skoko B. Jenkins S. (2020). Negotiating gender in the male-dominated skilled trades: a systematic literature review. Constr. Manag. Econ. 38, 894–916. doi: 10.1080/01446193.2020.1762906

[ref15] Bobbitt-Zeher D. (2011). Gender discrimination at work: connecting gender stereotypes, institutional policies, and gender composition of workplace. Gender Soc. 25, 764–786. doi: 10.1177/0891243211424741

[ref16] Born A. Ranehill E. Sandberg A. (2022). Gender and willingness to lead: does the gender composition of teams matter? Rev. Econ. Stat. 104, 259–275. doi: 10.1162/rest_a_00955

[ref17] Braddy P. W. Sturm R. E. Atwater L. Taylor S. N. McKee R. A. (2020). Gender bias still plagues the workplace: looking at derailment risk and performance with self–other ratings. Group Organ. Manag. 45, 315–350. doi: 10.1177/1059601119867

[ref18] Büyüköztürk Ş. Kılıç Çakmak E. Akgün Ö. E. Karadeniz Ş. Demirel F. (2012). Sampling methods. Istanbul/Turkey: Pegem Publishing House.

[ref19] Castaño A. M. Fontanil Y. García-Izquierdo A. L. (2019). “Why can’t I become a manager?” a systematic review of gender stereotypes and organizational discrimination. Int. J. Environ. Res. Public Health 16:1813. doi: 10.3390/ijerph16101813, PMID: 31121842 PMC6572654

[ref20] Cevher E. Öztürk U. C. (2015). A research on mobbing by women towards women in business life. J. Hum. Soc. Sci. Res. 4, 860–876. doi: 10.15869/itobiad.59306

[ref22] Childs S. (2012). Gender discrimination in the workplace. Doctoral dissertation: State University of New York Empire State College.

[ref23] Cibibin C. Leo I. (2022). The ‘queen bee syndrome’ in sports federations: an exploratory investigation of gender stereotypes in Italian female coaches. Sustain. For. 14:1596. doi: 10.3390/su14031596

[ref9001] Creswell J. (2013). Qualitative inquiry & research design: Choosing among five approaches.

[ref24] Creswell J. W. (2017). 30 essential skills for qualitative researchers. Ankara: Anı Publishing.

[ref25] Del Carpio L. Guadalupe M. (2022). More women in tech? Evidence from a field experiment addressing social identity. Manag. Sci. 68, 3196–3218. doi: 10.1287/mnsc.2021.4035, PMID: 19642375

[ref26] Derks B. Van Laar C. Ellemers N. (2016). The queen bee phenomenon: why women leaders distance themselves from junior women. Leadersh. Q. 27, 456–469. doi: 10.1016/j.leaqua.2015.12.007

[ref27] Dijksterhuis A. Nordgren L. F. (2006). A theory of unconscious thought. Perspect. Psychol. Sci. 1, 95–109. doi: 10.1111/j.1745-6916.2006.00007.x, PMID: 26151465

[ref9002] Dijksterhuis A. Strick M. (2016). A case for thinking without consciousness. Perspectives on Psychological Science, 11, 117–132. 26817729 10.1177/1745691615615317

[ref29] Drisko J. W. Maschi T. (2017). Content analysis. New York: Oxford University Press.

[ref30] Ellemers N. Van den Heuvel H. De Gilder D. Maass A. Bonvini A. (2004). The underrepresentation of women in science: differential commitment or the queen bee syndrome? Br. J. Soc. Psychol. 43, 315–338. doi: 10.1348/0144666042037999, PMID: 15479533

[ref31] Elo S. Kyngs H. (2008). The qualitative content analysis process. J. Adv. Nurs. 62, 107–115. doi: 10.1111/j.1365-2648.2007.04569.x 18352969

[ref32] Ely R. J. (1995). The power in demography: women's social constructions of gender identity at work. Acad. Manag. J. 38, 589–634. doi: 10.2307/256740

[ref33] Faniko K. Ellemers N. Derks B. (2016). Queen bees and alpha males: are succesul women more competitive than succesul men? Eur. J. Soc. Psychol. 46, 903–913. doi: 10.1002/ejsp.2198

[ref34] Foschi M. (1996). Double standards in the evaluation of men and women. Soc. Psychol. Q. 59, 237–254. doi: 10.2307/2787021

[ref35] Gabriel A. S. Butts M. M. Yuan Z. Rosen R. L. Sliter M. T. (2018). Fur-ther understanding incivility in the workplace: the effects of gender, agency, and communion. J. Appl. Psychol. 103:362. doi: 10.1037/apl0000289, PMID: 29239641

[ref36] Garcia-Retamero R. López-Zafra E. (2006). Prejudice against women in male-congenial environments: perceptions of gender role congruity in leadership. Sex Roles 55, 51–61. doi: 10.1007/s11199-006-9068-1

[ref38] Gomes Neto M. B. Grangeiro R. R. Esnard C. (2022). Academic women: a study on the queen bee phenomenon. RAM 23:eRAMG220211. doi: 10.1590/1678-6971/eramg220211.en

[ref39] Grangeiro R. D. R. Rezende A. T. Gomes Neto M. B. Carneiro J. S. Esnard C. (2022). Queen bee phenomenon scale: psychometric evidence in the Brazilian context. BAR 19:e210070. doi: 10.1590/1807-7692bar2022210070

[ref40] Gregory R. F. (2003). Women and workplace discrimination: Overcoming barriers to gender equality. Rutgers University Press.

[ref41] Harvey C. (2018). When queen bees attack women stop advancing: recognising and addressing female bullying in the workplace. Dev. Learn. Organ. 32, 1–4. doi: 10.1108/DLO-04-2018-0048, PMID: 35579975

[ref42] Hennink M. M. Kaiser B. N. Marconi V. C. (2017). Code saturation versus meaning saturation: how many interviews are enough? Qual. Health Res. 27, 591–608. doi: 10.1177/1049732316665344, PMID: 27670770 PMC9359070

[ref43] Holmes J. Marra M. Lazzaro-Salazar M. (2017). Negotiating the tall poppy syndrome in New Zealand workplaces: women leaders managing the challenge. Gender Lang. 11:236. doi: 10.1558/genl.31236

[ref44] Hussain M. (2022). Development and validation of queen bee syndrome perception inventory (QBSPI). Webology 19:4.

[ref9003] Ivy S. (2023). Unconscious intelligence in the skilled control of expert action. Journal of Consciousness Studies 30, 59–83.

[ref45] James R. Fisher J. R. Carlos-Grotjahn C. Boylan M. S. Dembereldash B. Demissie M. Z.. (2023). Gender bias and inequity holds women back in their conservation careers. Front. Environ. Sci. 10:2644. doi: 10.3389/fenvs.2022.1056751

[ref46] Johnson J. L. Adkins D. Chauvin S. (2020). A review of the quality indicators of rigor in qualitative research. Am. J. Pharm. Educ. 84:7120. doi: 10.5688/ajpe7120, PMID: 32292186 PMC7055404

[ref47] Kark R. Yacobovitz N. Segal-Caspi L. Kalker-Zimmerman S. (2020). Catty, bitchy, queen bee or sister? A review of competition among women in organizations from a paradoxical-coopetition perspective. J. Organ. Behav. 2020, 1–29. doi: 10.1002/job.2691

[ref48] Kato T. Kodama N. (2018). The effect of corporate social responsibility on gender diversity in the workplace: econometric evidence from Japan. Br. J. Ind. Relat. 56, 99–127. doi: 10.1111/bjir.12238

[ref9004] Klaus P. (2009). A sisterhood of workplace infighting. New York Times.

[ref49] Kremer H. Villamor I. Ormiston M. (2019). Princess bee effect: the reverse queen bee phenomenon. Acad. Manag. Proc. 2019:19515. doi: 10.5465/AMBPP.2019.253

[ref50] Leung L. (2015). Validity, reliability, and generalizability in qualitative research. J. Family Med. Prim. Care 4, 324–327. doi: 10.4103/2249-4863.161306, PMID: 26288766 PMC4535087

[ref51] Levitt H. M. Bamberg M. Creswell J. W. Frost D. M. Josselson R. Suárez-Orozco C. (2018). Journal article reporting standards for qualitative primary, qualitative meta-analytic, and mixed methods research in psychology: the APA publications and communications board task force report. Am. Psychol. 73, 26–46. doi: 10.1037/amp0000151, PMID: 29345485

[ref52] Maghni A. Afilal K. (2023). Female rivalry at work: case of Moroccan executives in the industrial sector. IJTM 1, 75–84. doi: 10.5281/zenodo.7688795

[ref53] Malos S. (2015). Overt stereotype biases and discrimination in the workplace: why haven’t we fixed this by now? Emp. Responsib. Rights J. 27, 271–280. doi: 10.1007/s10672-015-9264-7

[ref54] Malterud K. Siersma V. D. Guassora A. D. (2016). Sample size in qualitative interview studies: guided by information power. Qual. Health Res. 26, 1753–1760. doi: 10.1177/1049732315617444, PMID: 26613970

[ref55] Mancl A. C. Penington B. (2011). Tall poppies in the workplace: communication strategies used by envious others in response to successful women. Qual. Res. Rep. Commun. 12, 79–86. doi: 10.1080/17459435.2011.601701

[ref56] Martin A. E. Phillips K. W. (2017). What “blindness” to gender differences helps women see and do: implications for confidence, agency, and action in male-dominated environments. Organ. Behav. Hum. Decis. Process. 142, 28–44. doi: 10.1016/j.obhdp.2017.07.004

[ref57] Mavin S. (2006). Venus envy: problematizing solidarity behavior and queen bees. Women Manag. Rev. 21, 264–276. doi: 10.1108/09649420610666579

[ref58] Mavin S. Yusupova M. (2022). ‘I'm competitive with myself’: a study of women leaders navigating neoliberal patriarchal workplaces. Gend. Work. Organ. 30, 881–896. doi: 10.1111/gwao.12939

[ref59] Mert P. (2022). Özel Okullarda Çalışan Kadın Öğretmenlerin Kraliçe Arı Sendromu ile İlgili Görüşleri. Ahi Evran Üniversitesi Kırşehir Eğitim Fakültesi Dergisi 23, 1091–1118. doi: 10.29299/kefad.929504

[ref60] Mizrahi R. (2003). Hostility to the presence of women: why women undermine each other in the workplace and the consequences for title VII. Yale 113:1579.

[ref61] Mufti R. Moazzam A. Basit A. (2021). Queen bee syndrome a part of sexual politics or another gendered stereotype. J. Bus. Soc. Rev. Emerg. Econ. 7, 835–846. doi: 10.26710/jbsee.v7i4.2005

[ref62] Narcikara E. B. (2017). Psychological ownership in family business in the light of social identity theory. Int. J. Res. Soc. Sci. 7, 520–545.

[ref63] Narcikara E. B. (2018). Sosyal Kimlik Teorisi Perspektifiyle Kraliçe Ari Sendromu. Karadeniz Teknik Üniv. Sosyal Bilimler 8, 159–176.

[ref64] Netchaeva E. (2014). Developing a model of jealousy expression and manifestation in organizational settings within same-and mixed-sex triads: The University of Utah.

[ref65] Nieva V. F. Gutek B. A. (1981). Women and work: A psychological perspective: Praeger Publishers.

[ref66] Padavic I. Reskin B. F. (2002). Women and men at work: Pine Forge Press.

[ref67] Patton M. Q. (2002). Two decades of developments in qualitative inquiry: a personal, experiential perspective. Qual. Soc. Work. 1, 261–283. doi: 10.1177/1473325002001003636

[ref68] Peck J. A. (2021). The disproportionate impact of COVID-19 on women relative to men: a conservation of resources perspective. Gend. Work. Organ. 28, 484–497. doi: 10.1111/gwao.12597

[ref69] Phelan J. E. Moss-Racusin C. A. Rudman L. A. (2008). Competent yet out in the cold: shifting criteria for hiring reflect backlash toward Agen- tic women. Psychol. Women Q. 32, 406–413. doi: 10.1111/j.1471-6402.2008.0045

[ref70] Powell G. N. Butterfield D. A. (2003). Gender, gender identity, and aspirations to top management. Women Manag. Rev. 18, 88–96. doi: 10.1108/09649420310462361

[ref71] Raja B. I. Riaz S. (2022). Analyzing ‘queen bee syndrome’ in the context of women's leadership and managerial roles through empirical research. J. Dev. Soc. Sci. 3, 264–273. doi: 10.47205/jdss.2022(3-1)21

[ref73] Schieman S. McMullen T. (2008). Relational demography in the workplace and health: an analysis of gender and the subordinate–superordinate role-set. J. Health Soc. Behav. 49, 286–300. doi: 10.1177/002214650804900304 18771064

[ref74] Sheppard L. D. Aquino K. (2013). Much ado about nothing: observers’ problematization of women’s same-sex conflict at work. Acad. Manage. Perspect. 27, 52–62. doi: 10.5465/amp.2012.0005

[ref75] Sheppard L. D. Aquino K. (2017). Sisters at arms: a theory of female same-sex conflict and its problematization in organizations. J. Manag. 43, 691–715. doi: 10.1177/0149206314539348

[ref76] Sterk N. Meeussen L. Van Laar C. (2018). Perpetuating inequality: junior women do not see queen bee behavior as negative but are nonetheless negatively affected by it. Front. Psychol. 9:1690. doi: 10.3389/fpsyg.2018.01690, PMID: 30294289 PMC6159757

[ref77] Tabassum N. Nayak B. S. (2021). Gender stereotypes and their impact on women’s career progressions from a managerial perspective. IIM Kozhikode Soc. Manag. Rev. 10, 192–208. doi: 10.1177/2277975220975513

[ref9005] Vara-Horna A. A. Asencios-Gonzalez Z. B. Quipuzco-Chicata L. Díaz-Rosillo A. (2022). Are companies committed to preventing gender violence against women? The role of the manager’s implicit resistance. Social Sciences, 12:12.

[ref78] Vial A. C. Napier J. L. Brescoll V. L. (2016). A bed of thorns: female leaders and the self-reinforcing cycle of illegitimacy. Leadersh. Q. 27, 400–414. doi: 10.1016/j.leaqua.2015.12.004

[ref79] Webber G. R. Giuffre P. (2019). Women's relationships with women at work: barriers to solidarity. Sociol. Compass 13:e12698. doi: 10.1111/soc4.12698

[ref80] Wright T. (2016). Women's experience of workplace interactions in male-dominated work: the intersections of gender, sexuality and occupational group. Gender Work Organ. 23, 348–362. doi: 10.1111/gwao.12074

[ref81] Wuertele R. (2017) The influence of the queen bee syndrome on the attitudes, behaviors, and emerging leadership styles of the millennials.

[ref82] Yildirim T. M. Eslen-Ziya H. (2021). The differential impact of COVID-19 on the work conditions of women and men academics during the lockdown. Gender Work Organ. 28, 243–249. doi: 10.1111/gwao.12529, PMID: 32904915 PMC7461380

[ref83] Yin R. K. (2009). “How to do better case studies” in The SAGE handbook of applied social research methods, 254–282.

[ref85] Zhao S. Maw-Der-Foo Q. (2016). Queen bee syndrome – the real reason women do not promote women, Center for Creative Leadership [Online]. Available at: https://www.ccl.org/wp-content/uploads/2016/09/Queen-Bee-Syndrome.pdf. (Accessed May 18, 2018).

